# Chronic Kidney Disease Has a Graded Association with Death and Cardiovascular Outcomes in Stable Coronary Artery Disease: An Analysis of 21,911 Patients from the CLARIFY Registry

**DOI:** 10.3390/jcm9010004

**Published:** 2019-12-18

**Authors:** Emmanuelle Vidal-Petiot, Nicola Greenlaw, Paul R. Kalra, Xavier Garcia-Moll, Jean-Claude Tardif, Ian Ford, Jose Zamorano, Roberto Ferrari, Michal Tendera, Kim M. Fox, Philippe Gabriel Steg, on behalf of the CLARIFY investigators

**Affiliations:** 1 Université de Paris, Paris, France; emmanuelle.vidal-petiot@aphp.fr; 2 Physiology Department, Assistance Publique-Hôpitaux de Paris, Hôpital Bichat, 75018 Paris, France; 3 Institut nationale de la santé et de la recherché médicale (INSERM) U1149, Centre de Recherche sur l’Inflammation, 75018 Paris, France; 4 Robertson Centre for Biostatistics, University of Glasgow, Glasgow G12 8QQ, UK; Nicola.Greenlaw@glasgow.ac.uk (N.G.); Ian.Ford@glasgow.ac.uk (I.F.); 5 Portsmouth Hospitals NHS Trust, Portsmouth PO6 3LY, UK; paulkalra@doctors.org.uk; 6 Hospital Santa Creu i Sant Pau, 08041 Barcelona, Spain; XGarcia-Moll@santpau.cat; 7 Montreal Heart Institute, Université de Montreal, Montreal, QC H1T1C8, Canada; Jean-Claude.Tardif@icm-mhi.org; 8 University Hospital Ramon y Cajal, 28040 Madrid, Spain; zamorano@secardiologia.es; 9 Centro Cardiologico Universitario di Ferrara, University of Ferrara, 44124 Cona, FE, Italy; roberto.ferrari@unife.it; 10 Maria Cecilia Hospital, GVM Care & Research, 48033 Cotignola, RA, Italy; 11 Department of Cardiology and Structural Heart Disease, School of Medicine in Katowice, Medical University of Silesia, 40-055 Katowice, Poland; michal.tendera@gmail.com; 12 National Heart and Lung Institute, Imperial College, Royal Brompton Hospital, London SW3 6NP, UK; kim.fox@imperial.ac.uk; 13 Cardiology Department, Assistance Publique-Hôpitaux de Paris, Hôpital Bichat, French Alliance for Cardiovascular Trials, INSERM U1148, 75018 Paris, France

**Keywords:** chronic kidney disease, chronic coronary artery disease, CLARIFY registry

## Abstract

Chronic kidney disease (CKD) is associated with an increased cardiovascular risk in a broad spectrum of populations. However, the risk associated with a reduced estimated glomerular filtration rate (eGFR) in patients with stable coronary artery disease receiving standard care in the modern era, independently of baseline cardiovascular disease, risk factors, and comorbidities, remains unclear. We analyzed data from 21,911 patients with stable coronary artery disease, enrolled in 45 countries between November 2009 and July 2010 in the CLARIFY registry. Patients with abnormal renal function were older, with more comorbidities, and received slightly lower—although overall high—rates of evidence-based secondary prevention therapies than patients with normal renal function. The event rate of patients with CKD stage 3b or more (eGFR <45 mL/min/1.73 m^2^) was much higher than that associated with any comorbid condition. In a multivariable adjusted Cox proportional hazards model, lower eGFR was independently associated with a graded increased risk of cardiovascular mortality, with adjusted HRs (95% CI) of 0.98 (0.81–1.18), 1.31 (1.05–1.63), 1.77 (1.38–2.27), and 3.12 (2.25–4.33) for eGFR 60–89, 45–59, 30–44, and <30 mL/min/1.73 m^2^, compared with eGFR ≥90 mL/min/1.73 m^2^. A strong graded independent relationship exists between the degree of CKD and cardiovascular mortality in this large cohort of patients with chronic coronary artery disease, despite high rates of secondary prevention therapies. Among clinical risk factors and comorbid conditions, CKD stage 3b or more is associated with the highest cardiovascular mortality.

## 1. Introduction

Chronic kidney disease (CKD) is a well-recognized risk factor for mortality and cardiovascular disease. This increased risk has been noted in general population cohorts [1,2,3,4,5], in cohorts at high risk for, or with established, cardiovascular disease [4,6] and in patients with heart failure [7]. However, few data are available in the specific population of patients with stable coronary artery disease, especially in a contemporary cohort including patients largely treated with drugs associated with prognostic benefit [8] and without severe heart failure at inclusion.

We used data from the global prospective observational longitudinal registry of patients with chronic coronary artery disease (CLARIFY) to compare the risk associated with different stages of CKD with that associated with other risk factors and comorbid conditions. In addition, we assessed whether reduced renal function is a risk factor, independent of other established risk factors and comorbidities, in patients with chronic coronary artery disease treated according to standard care in the modern era.

## 2. Methods

### 2.1. Study Design and Participants

Clarify is an international, prospective, observational, longitudinal registry of outpatients with chronic coronary artery disease (ISRCTN43070564; www.clarifyregistry.com). The study rationale and methods have been published elsewhere [9,10]. Briefly; 32,703 patients were enrolled in 45 countries in Africa, Asia, Australia, Europe, the Middle East, and North, Central, and South America between November 2009 and June 2010. Eligible patients had chronic coronary artery disease proven by a history of at least one of the following: Documented myocardial infarction more than three months before enrolment; angiographic demonstration of coronary stenosis of more than 50%; chest pain with evidence of myocardial ischemia (stress electrocardiogram, stress echocardiograph, or myocardial perfusion imaging); or coronary artery bypass graft or percutaneous coronary intervention performed more than three months before enrolment. These criteria were not mutually exclusive. Exclusion criteria were hospital admission for cardiovascular reasons (including revascularization) in the past 3 months; planned revascularization; conditions hampering participation over 5-year follow-up, such as limited cooperation, inability to provide informed consent, serious non-cardiovascular disease, or conditions interfering with life expectancy (e.g., cancer; drug abuse); or other severe cardiovascular diseases such as advanced heart failure, severe valve disease, or history of valve repair or replacement. CKD, per se, was not an exclusion criterion. To ensure that the study population was representative of chronic coronary artery disease outpatients, recruitment of sites and subjects was based on a predefined selection of physicians (cardiologists as well as office-based primary care physicians and physicians based in hospitals with outpatient clinics) by national coordinators, using the best available epidemiological data in each country reflecting the burden of coronary artery disease; this was done in an attempt to provide a distribution of physicians across regions and locations (i.e., urban; suburban; or rural areas), mimicking the epidemiological patterns in each country. In each practice; patient recruitment was restricted over a brief period to achieve near-consecutive patient enrolment in order to avoid selection bias. The study was conducted in accordance with the principles in the Declaration of Helsinki and local ethical approval was obtained in all countries prior to recruitment. All patients gave written informed consent before any data acquisition was performed. The study was registered at ISRCTN as ISRCTN43070564.

### 2.2. Data Collection

The investigators completed standardized case report forms at baseline and at a yearly patient visit (plus or minus three months) for up to 5 years. In addition, telephone contact with the patient, a designated relative or contact, or their physician was attempted in the 6-month interim between the yearly visits. At each yearly visit, symptoms, clinical examination, results of the main recent clinical and biological tests, treatment, and clinical outcomes were recorded. Patients were treated according to usual clinical practice at each institution, with no specific tests or therapies defined in the study protocol. Where applicable, registries could be used to retrieve the vital status. Events were accepted as reported by physicians and were not adjudicated. Events were accepted as reported by physicians following the detailed requirement of the case reports forms and were not adjudicated. However, to ensure data quality, onsite monitoring visits were carried out in 5% of randomly selected centres with source verification of all events and of 100% of the collected data, centralized verification of the electronic case report forms were performed for completeness, consistency, and accuracy, and regular telephone contacts with investigators were done to limit missing data and loss to follow-up.

Baseline estimated glomerular filtration rate (eGFR) was derived from the creatinine-derived chronic kidney disease Epidemiology Collaboration (CKD-EPI) equation [11], and analyzed in five categories: <30, 30–44, 45–59, 60–89, and ≥90 mL/min/1.73 m^2^, the latter being used as reference. Analyses were also conducted using eGFR as a continuous variable, with values above 90 mL/min/1.73 m^2^ truncated at this threshold. Results were provided per 5 unit decrease in eGFR below this threshold and scaled at 90 mL/min/1.73 m^2^.

A flow diagram of the study population is shown in eFigure in the Supplementary Materials; 325 patients had an incomplete 5-year follow-up, 10,407 patients had incomplete sets of variables required to calculate eGFR (mainly ethnicity, which local ethics committees did not authorize collecting in France and Portugal, and creatinine values), and patients with baseline eGFR below 10 mL/min/1.73 m^2^ (*n* = 60) were excluded, leaving a total of 21,911 patients in the present analysis.

### 2.3. Study Outcomes

Pre-specified outcomes of interest were cardiovascular death (primary outcome), all-cause death, myocardial infarction (fatal or not), stroke (fatal or not), and hospital admission for heart failure (secondary outcomes). Cardiovascular death was defined as death from myocardial infarction or stroke, any sudden death including unobserved and unexpected death (while sleeping) unless proven otherwise by autopsy, death ascribed to heart failure, death following cardiac or vascular procedure/operation, death due to ruptured aneurysm or pulmonary embolism, death due to amputation (except for trauma or malignancy), and death that could not be definitely ascribed to non-cardiovascular death.

### 2.4. Statistical Analysis

Continuous variables were presented as mean ± standard deviation or median (interquartile range), depending on the distribution of the data; categorical data were presented as count and percentage. Comparisons between eGFR categories were performed using one-way ANOVA, or Kruskal-Wallis test for continuous variables as appropriate and Chi-Squared tests for categorical data.

Event rates and corresponding 95% confidence intervals (CI) for the primary outcome and each of the secondary outcomes were provided per 1000 patient-years, using the date of event or censoring date. If multiple events were recorded, the event rate was calculated using the date of first event.

Cox proportional hazards models were used to assess the association between eGFR, in categories or as a continuous variable, and outcomes. In addition to crude hazard ratios (HRs), adjusted HRs were estimated after adjustment for potential confounding factors, selected a priori as potential confounders, namely age, sex, geographical region, smoking status, diabetes, body-mass index, treated hypertension, baseline systolic blood pressure, low-density and high-density lipoprotein cholesterol levels, previous myocardial infarction, previous percutaneous coronary intervention, previous coronary artery bypass grafting, number of diseased coronary vessels at baseline, peripheral artery disease at baseline, previous stroke or transient ischemic attack, previous hospital admission for (or symptoms of) heart failure, left ventricular ejection fraction, atrial fibrillation or flutter, and baseline drugs (any antiplatelet, statins, angiotensin-converting enzyme inhibitors, angiotensin-receptor blockers, and beta-blockers).

Data were analyzed as recorded without imputation for missing data. Adjustment variables were analyzed including a category for missing data to minimize the loss of data in the analysis.

Interaction between eGFR and diabetes, treated hypertension, and age (< or ≥60 years) were tested for the categorical and continuous analyses of eGFR.

The statistical analysis was done with SAS (version 9.3).

## 3. Results

A total of 21,911 patients with chronic coronary artery disease and available baseline eGFR were included in the analysis. Baseline characteristics of the patients, in the total study population and by category of baseline eGFR, are reported in Table 1. Mean age was 63.9 ± 10.4 years, 16,941 (77%) were men, 15,731 (72%) had treated hypertension, 6646 (30%) had diabetes, and 2788 (13%) and 10,261 (47%) were current and former smokers, respectively. Mean eGFR was 76 ± 19 mL/min/1.73 m^2^.

Compared with patients with eGFR ≥ 90 mL/min/1.73 m^2^, those with a lower eGFR were older, more likely to be female, have diabetes and hypertension, and less likely to be current smokers. They were less likely to have undergone percutaneous coronary intervention, and more likely to have undergone coronary artery bypass grafting. They had a higher prevalence of peripheral artery disease, heart failure, previous stroke, and atrial fibrillation or flutter. They were less likely to receive antiplatelet agents, lipid lowering drugs, and angiotensin-converting enzyme inhibitors, but more likely to receive angiotensin II receptor blockers. The rate of prescription of any renin-angiotensin system blocker was lowest (70.4%) in patients with eGFR <30 mL/min/1.73 m^2^.

After a median follow-up of 5 years (4.82; 5.10), 1837 patients died (1158 of cardiovascular cause), 829 patients had a myocardial infarction (fatal or not), 493 had a stroke (fatal or not), and 1279 were hospitalized for heart failure.

**Figure 1:**
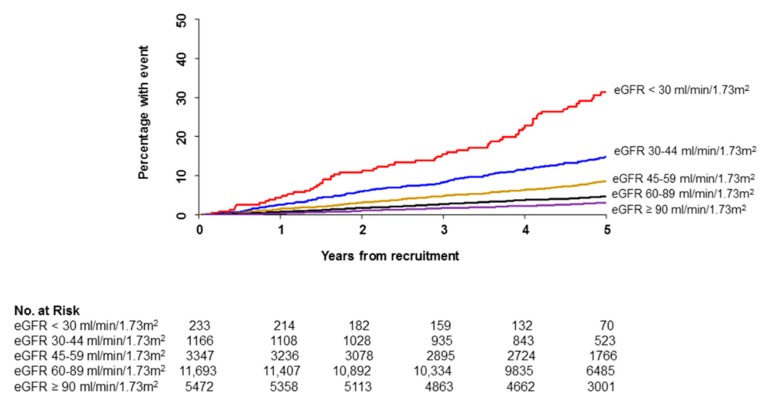
Kaplan-Meier plots showing cumulative incidence of cardiovascular death by eGFR category.

The cumulative incidence of cardiovascular death increased with decreasing eGFR (Figure 1).

**Figure 2:**
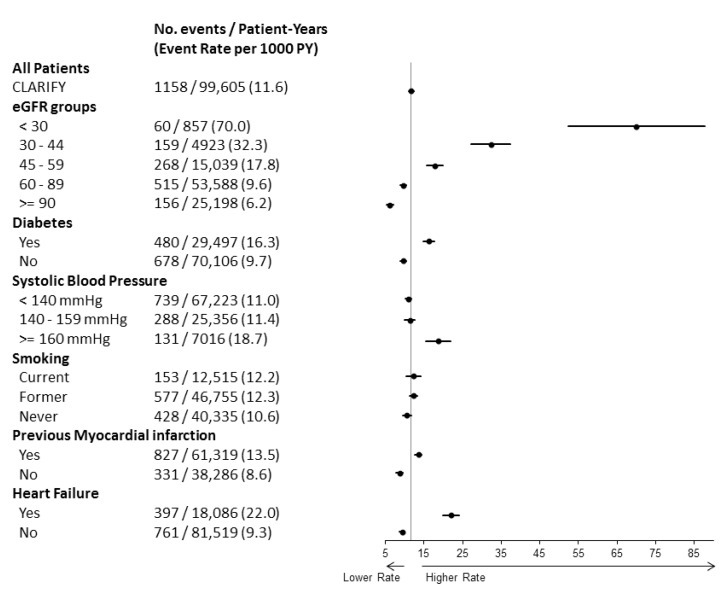
Event rates for cardiovascular death. Unadjusted rates (95% CI) per 1000 patient-years for cardiovascular death are indicated in the total study population, by category of eGFR, diabetic status, systolic blood pressure level, smoking status, previous myocardial infarction, and history of heart failure.

Compared to major risk factors for coronary artery disease and prior cardiovascular history (such as previous myocardial infarction or heart failure), CKD stage 3b or more was, by far, the one associated with the highest increase in the unadjusted risk of cardiovascular death (Figure 2).

Event rates, and crude and adjusted HRs for all outcomes, for each eGFR category, are reported in Table 2. Unadjusted rates of cardiovascular mortality were 6.2, 9.6, 17.8, 32.3, and 70.0 per 1000 patient-years in patients with eGFR ≥ 90, 60–89, 45–59, 30–44, and <30 mL/min/1.73 m^2^. Even after multiple adjustments for baseline cardiovascular disease, risk factors, comorbidities, and drugs, a lower eGFR was associated with increased risk of cardiovascular death, with adjusted HRs (95% CI) of 0.98 (0.81–1.18), 1.31 (1.05–1.63), 1.77 (1.38–2.27), and 3.12 (2.25–4.33), for eGFR 60–89, 45–59, 30–44, and <30 mL/min/1.73 m^2^, as compared with eGFR ≥ 90 mL/min/1.73 m^2^. The adjusted risk was also significantly increased as early as stage 3a CKD (eGFR 45–59 mL/min/1.73 m^2^) for all-cause death, with adjusted HRs (95% CI) of 0.99 (0.85–1.15), 1.23 (1.03–1.46), 1.72 (1.41–2.10), and 2.96 (2.27–3.86) for eGFR 60–89, 45–59, 30–44, and <30 mL/min/1.73 m^2^, as compared with eGFR ≥ 90 mL/min/1.73 m^2^, and for admission for heart failure, with adjusted HRs (95% CI) of 1.10 (0.93–1.29), 1.36 (1.12–1.66), 2.08 (1.65–2.63), and 2.11 (1.44–3.11) for eGFR 60–89, 45–59, 30–44, and <30 mL/min/1.73 m^2^, as compared with eGFR ≥ 90 mL/min/1.73 m^2^. Although crude risk increased as eGFR decreased for myocardial infarction and stroke, adjusted HRs were only significantly increased for myocardial infarction when eGFR was below 30 mL/min/1.73 m^2^.

When eGFR was analyzed as a continuous variable, worsening of eGFR below 90 mL/min/1.73 m^2^ was significantly associated with all outcomes, with adjusted HRs (95% CI) of 1.079 (1.060–1.099), 1.073 (1.057–1.088), 1.029 (1.005–1.053), 1.033 (1.004–1.063), and 1.061 (1.042–1.080) for cardiovascular death, all-cause death, myocardial infarction, stroke, and hospital admission for heart failure, respectively (Table 3).

Interaction analyses are presented in eTable in the Supplementary Materials. No significant interaction was found between CKD and diabetes, treated hypertension, or age when eGFR was analyzed in five categories. When eGFR was analyzed as a continuous variable, a significant interaction was observed between eGFR and history of hypertension. Indeed, a decreasing eGFR was associated with an increased risk of myocardial infarction and with an increased risk of stroke only in patients with hypertension (*p*-values for interaction 0.0090 and 0.0313 for myocardial infarction and stroke, respectively). 

## 4. Discussion

In this large international contemporary registry of patients with coronary artery disease, a reduced eGFR was associated with a gradual increase in the risks of mortality and adverse cardiovascular events. This increased risk appeared as early as CKD stage 3a (eGFR 45–59 mL/min/1.73 m^2^) for both cardiovascular and total mortality, and persisted after multiple adjustments for potential confounding factors, including cardiovascular risk factors, overt cardiovascular disease at baseline, comorbidities, and medication. In addition, the large size of the cohort allowed us to reliably estimate the risks associated with CKD, compared to those associated with diabetes, elevated systolic blood pressure, smoking, and previous myocardial infarction or heart failure in patients with coronary artery disease treated in routine practice. CKD stage 3b or more, as estimated from the CKD-EPI equation [11,12], was a marker of substantially increased risk in patients with coronary artery disease, far higher than that associated with all above-mentioned risk factors and conditions.

In the present analysis, the risk associated with eGFR 60–89 mL/min/1.73 m^2^ did not differ from that associated with eGFR ≥ 90mL/min/1.73 m^2^. Accordingly, for GFR values of 60 mL/min/1.73 m^2^ or more, the GFR value, per se, does not define CKD in the absence of markers of kidney damage such as albuminuria or morphological abnormalities [13].

Below 60 mL/min/1.73 m^2^, prior studies have generally shown an association between a lower eGFR and adverse cardiovascular outcomes and mortality [14], although data are not all consistent, especially regarding the threshold of eGFR below which risk increases.

In the REACH registry of patients at very high cardiovascular risk, Dumaine et al. found that severe CKD (as estimated by creatinine clearance below 30 mL/min/1.73 m^2^, hence a true GFR below this threshold) was associated in increased risks of mortality, myocardial infarction, and death, whereas no significantly increased risk was found above this value [6]. Similarly, several studies conducted in community-based cohorts found no significant association between moderate CKD and death or cardiovascular disease [15,16]. In contrast, in a meta-analysis of general population cohorts, Matsushita et al. found that mortality was unrelated to eGFR above 75 mL/min/1.73 m^2^, and increased below this value [3]. Likewise, in 15,582 subjects from the Atherosclerosis Risk in Communities (ARIC) study, an eGFR between 60 and 89 mL/min/1.73 m^2^ was associated with an increased risk of atherosclerotic cardiovascular disease compared with an eGFR of 90 to 150 mL/min/1.73 m^2^ [2].

As previously observed in numerous other studies [17], the magnitude of the risk associated with a lower eGFR diminished after adjustment, indicating that demographic and clinical characteristics of patients with CKD, including older age, higher blood pressure [18], and prior cardiovascular disease [19], contribute in part to the association between CKD and risks of death and cardiovascular disease. Comorbidities ought to be taken into account when assessing renal and cardiovascular risks of patients with CKD [20]. In addition, although overall rates of secondary prevention therapies were high in the CLARIFY population, patients with CKD received suboptimal treatment [8]. The proportion of patients receiving antiplatelets, lipid-lowering drugs, and beta-blockers decreased from 97.8% to 88.8%, 94.4% to 91.4%, and 78.3% to 72.1%, respectively, in patients with eGFR ≥ 90 compared with those with eGFR <30 mL/min/1.73 m^2^. Patients with CKD stage 3 received the highest rate of renin-angiotensin system blockers (80.1% for CKD stage 3a and 79.7% for CKD stage 3b), but this rate decreased to 70.4% in patients with eGFR <30mL/min/1.73 m^2^. In addition to the higher burden of comorbidities [20], suboptimal secondary prevention may contribute in part to the higher event rates observed with more advanced CKD [21,22,23].

However, a lower eGFR was still strongly associated with adverse outcomes after adjustment for risk factors, baseline cardiovascular disease, comorbidities, and medications. Multiple pathophysiological explanations have been proposed in favour of a causal link underlying the adverse cardiovascular profile of CKD patients [1,24]. These include increased intravascular calcium phosphate deposition and coronary artery calcification, [25,26,27] arterial stiffness, enhanced coagulability, endothelial dysfunction, increased subclinical inflammation, [28] volume overload [29], and left ventricular remodeling and dysfunction [30,31]. In addition, patients with CKD undergoing percutaneous coronary intervention for an acute coronary syndrome have been shown to have less complete revascularization, a condition associated with a poorer prognosis [32].

Our results draw attention to the particularly high risk associated with CKD in patients with coronary artery disease. Whether or not CKD is a “coronary artery disease risk equivalent” (i.e., the risk associated with CKD in patients without coronary artery disease at baseline is equivalent to that associated with established coronary artery disease) is debated [17,33], but the present study shows that in patients with established chronic coronary artery disease, CKD markedly and dose-dependently further increases the risks for total and cardiovascular mortality and cardiovascular events. This increased risk is similar to that conferred by diabetes or a systolic blood pressure ≥160 mmHg for CKD stage 3a, and much higher than that of any risk factor or comorbid condition, including a history of heart failure, for CKD stage 3b or more. These results obtained in patients with coronary artery disease are line with those obtained in the general population by Tonelli et al., who showed a higher cardiovascular risk in patients with eGFR <60 and even more so <45 mL/min/1.73 m^2^ than in those with diabetes [17].

These results strongly support integrating eGFR in risk assessment and treatment targets for secondary prevention in patients with coronary artery disease. Patients with eGFR below 60 mL/min/1.73 m^2^ need to be monitored carefully and clinicians should attempt to optimize secondary prevention throughout follow-up. Prevention of end-stage renal disease is very important, but not the only goal in these patients who require effective cardiovascular intervention to reduce the exponential cardiovascular burden associated with decreasing eGFR. Patients are more likely to die or suffer adverse cardiovascular events than develop end-stage renal disease [12,14].

The prevalence of diabetes in our study population was 30%. Interestingly, no interaction was found between eGFR and diabetes, for any of the studies outcomes. This is in line with the meta-analysis by Fox et al. which showed, in thirty combined general population and high-risk cohorts, that the relative increases in risks of total and cardiovascular mortality with lower eGFR were the same in patients with and without diabetes [34]. Altogether, this supports the use of similar thresholds for diagnosis and classification of CKD in the presence or absence of diabetes. It’s noteworthy that patients with both CKD and diabetes, or at high risk for new onset diabetes, are at extremely high risk due to the combination of both conditions [35].

The analysis of eGFR as a continuous variable revealed a significant interaction between worsening renal function and a history of treated hypertension for the outcomes myocardial infarction and stroke (significant effect only in patients with hypertension). However, no interaction with hypertension was observed for the other outcomes, including cardiovascular mortality. In contrast, in the study by Mahmoodi et al. [4], the relative risk of mortality associated with a lower eGFR was stronger in non-hypertensive than hypertensive patients. Overall, the presence of CKD should be considered a relevant additional risk factor for cardiovascular mortality, irrespective of hypertension and diabetes status.

Strengths of our study include that patients were recruited from 43 countries, and treated according to routine clinical practice, with detailed and source-verified information on risk factors and comorbidities as well as outcome identification, and thereby reflect worldwide epidemiology of coronary artery disease patients, and are more generalizable than results obtained in the highly selected populations from most randomized clinical trials. These data are unique in a contemporary cohort of patients with chronic coronary artery disease and provide important data on the risk associated with CKD, versus that associated with other risks factors including diabetes, in this specific population. In addition, the large sample size allowed adjusting for multiple potential confounders, which is very important in the case of CKD patients who suffer multiple comorbidities.

Our study has several limitations. Due to restrictions preventing collection of ethnicity data in some European countries, eGFR based on the CKD-EPI equation was not available in the entire CLARIFY population. Another limitation, inherent to the nature of a registry, was that serum creatinine concentration measurement was not standardized across centres. In addition, proteinuria was not collected in the CLARIFY registry, so we could not analyze the expected even higher risk in patients with both CKD and proteinuria [3,5].

In conclusion, eGFR <60 mL/min/1/73 m^2^ was associated with a gradually increasing risk of all-cause and cardiovascular mortality, independently of multiple potential confounders. Even moderately reduced eGFR should draw at least as much attention as well-recognized risk factors and high-risk conditions such as diabetes, uncontrolled hypertension, and previous myocardial infarction. These findings are unique in a population with chronic coronary artery disease and underscore the high cardiovascular risk associated with CKD, especially of stage 3 or more, suggesting the clinical and public health importance of identifying and treating risk factors for cardiovascular disease in these patients.

## Figures and Tables

**Table 1:** Demographic and baseline characteristics of the patients, for the total population (*n* = 21,911) and by estimated glomerular filtration rate (eGFR) subgroup.

Parameter	Number of Patients	Total Population	GFR Subgroups (in mL/min/1.73 m^2^)					*p*-Value
			<30	30–44	45–60	60–89	≥90	
		(*n* = 21,911)	(*n* = 233)	(*n* = 1166)	(*n* = 3347)	(*n* = 11,693)	(*n* = 5472)	
Age (years)	21,911	63.9 (10.4)	73.1 (9.5)	71.9 (9.0)	69.84 (8.7)	64.7 (9.5)	56.5 (8.8)	<0.0001
Men	21,911	16,941 (77.3)	141 (60.5)	735 (63.0)	2254 (67.3)	9223 (78.9)	4588 (83.9)	<0.0001
Body Mass Index (kg/m^2^)	21,897	27.5 [24.9, 30.5]	27.2 [24.2, 30.8]	27.6 [24.8, 30.9]	27.5 [24.9, 30.6]	27.4 [25, 30.4]	27.5 [24.9, 30.8]	0.2438
Diabetes	21,909	6646 (30.3)	131 (56.2)	521 (44.7)	1143 (34.2)	3231 (27.6)	1620 (29.6)	<0.0001
Smoking status	21,911							
Current	-	2788 (12.7)	14 (6.0)	69 (5.9)	248 (7.4)	1431 (12.2)	1026 (18.8)	<0.0001
Former	-	10,261 (46.8)	112 (48.1)	508 (43.6)	1486 (44.4)	5612 (48.0)	2543 (46.5)	-
Never	-	8862 (40.4)	107 (45.9)	589 (50.5)	1613 (48.2)	4650 (39.8)	1903 (34.8)	-
Treated hypertension	21,910	15,731 (71.8)	211 (90.6)	984 (84.4)	2621 (78.3)	8332 (71.26)	3583 (65.5)	<0.0001
Systolic blood pressure (mm Hg)	21,908	131 (17)	133 (20)	133 (18)	133 (18)	131 (17)	129 (16)	<0.0001
Diastolic blood pressure (mm Hg)	21,908	77 (10)	74 (12)	76 (11)	77 (10)	77 (10)	78 (10)	<0.0001
Heart Rate (beats/minute)	21,910	69 (11)	70 (12.3)	69 (12)	69 (11)	68 (11)	69 (11)	<0.0001
Myocardial Infarction	21,911	13,550 (61.8)	136 (58.4)	735 (63.0)	1987 (59.4)	7187 (61.5)	3505 (64.05)	0.0001
Percutaneous coronary intervention	21,911	12,389 (56.5)	108 (46.34)	579 (49.7)	1700 (50.8)	6549 (56.0)	3453 (63.1)	<0.0001
Coronary artery bypass graft surgery	21,911	5298 (24.2)	89 (38.2)	328 (28.1)	967 (28.9)	2880 (24.6)	1034 (18.9)	<0.0001
Peripheral artery disease	21,909	2095 (9.6)	44 (18.9)	180 (15.4)	418 (12.5)	1065 (9.1)	388 (7.1)	<0.0001
Transient Ischemic Attack	21,910	679 (3.1)	10 (4.3)	75 (6.4)	176 (5.3)	306 (2.6)	112 (2.05)	<0.0001
Stroke	21,910	940 (4.3)	26 (11.2)	105 (9.0)	202 (6.0)	481 (4.1)	126 (2.3)	<0.0001
Atrial fibrillation/Flutter	21,911	1582 (7.2)	34 (14.6)	157 (13.5)	379 (11.3)	831 (7.1)	181 (3.3)	<0.0001
Hospitalization for heart failure	21,911	1104 (5.00)	48 (20.6)	143 (12.3)	236 (7.1)	504 (4.3)	173 (3.2)	<0.0001
Symptoms of heart failure	21,911							
None	-	18,348 (83.7)	184 (79.0)	915 (78.5)	2700 (80.7)	9861 (84.3)	4688 (85.67)	<0.0001
NYHA Class II	-	2969 (13.6)	37 (15.9)	201 (17.2)	514 (15.4)	1557 (13.3)	660 (12.06)	-
NYHA Class III	-	594 (2.7)	12 (5.2)	50 (4.3)	133 (4.0)	275 (2.4)	124 (2.27)	-
Left Ventricular Ejection Fraction (%)	15,731	55.6 (11.2)	49.3 (14.6)	52.3 (12.6)	54.8 (11.8)	56.1 (10.9)	56.03 (10.44)	<0.0001
HbA1C (%)	6638	6.84 (1.82)	7.24 (1.67)	7.09 (1.54)	6.92 (2.69)	6.74 (1.6)	6.89 (1.6)	<0.0001
Creatinine (mmol/L)	21,911	0.088 [0.076, 0.101]	0.198 [0.186, 0.226]	0.141 [0.125, 0.156]	0.112 [0.1, 0.122]	0.088 [0.08, 0.097]	0.071 [0.062, 0.079]	<0.0001
eGFR (mL/min/1.73 m^2^)	21,911	76 (19)	25 (4)	39 (4)	54 (4)	76 (9)	99 (7)	-
Total Cholesterol (mmol/L)	20,843	4.3 [3.6, 5]	4.2 [3.6, 4.9]	4.2 [3.5, 5]	4.3 [3.6, 5.1]	4.3 [3.7, 5]	4.2 [3.6, 5]	0.0027
HDL-cholesterol (mmol/L)	18,513	1.1 [1.0, 1.4]	1.0 [0.9, 1.3]	1.1 [0.9, 1.3]	1.1 [1.0, 1.4]	1.1 [1.0, 1.4]	1.1 [0.9, 1.3]	<0.0001
LDL-cholesterol (mmol/L)	17,505	2.3 [1.9, 2.9]	2.3 [1.7, 2.9]	2.2 [1.8, 2.8]	2.3 [1.9, 2.9]	2.4 [1.9, 3.0]	2.4 [1.9, 3.0]	0.0024
Fasting Triglycerides (mmol/L)	19,262	1.4 [1.0, 2.0]	1.6 [1.1, 2.0]	1.5 [1.1, 2.1]	1.5 [1.1, 2.0]	1.4 [1.0, 1.9]	1.4 [1.0, 2.0]	<0.0001
Baseline medication								
Aspirin	21,910	19,560 (89.3)	180 (77.3)	947 (81.2)	2867 (85.7)	10,460 (89.5)	5106 (93.3)	<0.0001
Thienopyridine	21,902	5650 (25.8)	76 (32.6)	302 (25.9)	795 (23.8)	2855 (24.4)	1622 (29.7)	<0.0001
Any antiplatelet agent	21,911	20,895 (95.4)	207 (88.8)	1055 (90.5)	3118 (93.2)	11,166 (95.5)	5349 (97.8)	<0.0001
Lipid-lowering drugs	21,911	20,470 (93.4)	213 (91.4)	1081 (92.7)	3074 (91.8)	10,938 (93.5)	5164 (94.4)	<0.0001
Beta-Blockers	21,910	16,625 (75.9)	168 (72.1)	893 (76.6)	2512 (75.1)	8766 (75.0)	4286 (78.3)	<0.0001
Calcium antagonists	21,909	5956 (27.2)	96 (41.2)	372 (31.9)	1065 (31.8)	3155 (27.0)	1268 (23.2)	<0.0001
Angiotensin-converting enzyme inhibitors	21,910	11,408 (52.1)	82 (35.2)	534 (45.8)	1692 (50.6)	6125 (52.4)	2975 (54.4)	<0.0001
Angiotensin II receptor antagonists	21,909	5873 (26.8)	90 (38.6)	445 (38.2)	1074 (32.1)	3067 (26.2)	1197 (21.9)	<0.0001
Any renin-angiotensin system blocker	21,910	16,843 (76.9)	164 (70.4)	929 (79.7)	2680 (80.1)	8975 (76.8)	4095 (74.9)	<0.0001
Diuretics	21,910	6492 (29.6)	156 (67.0)	633 (54.3)	1380 (41.2)	3193 (27.3)	1130 (20.7)	<0.0001

Data are mean (SD), median (IQR), or number (%). Some percentages do not add up to 100 because of rounding. eGFR = Glomerular Filtration Rate estimated from the CKD-EPI equation; NYHA = New York Heart Association (NYHA) Functional Classification; HDL-cholesterol = high-density lipoprotein cholesterol; LDL-cholesterol = low-density lipoprotein cholesterol.

**Table 2:** Event rates and crude and adjusted hazard ratios by GFR subgroups.

	GFR Subgroups (in mL/min/1.73 m^2^)					*p*-Value
	<30 (*n* = 233)	30–44 (*n* = 1166)	45–59 (*n* = 3347)	60–89 (*n* = 11693)	≥ 90 (*n* = 5472)	
**Cardiovascular death**						
number of events	60	159	268	515	156	
event rate (per 1000 P-Y)	70.0 (52.3–87.7)	32.3 (27.3–37.3)	17.8 (15.7–20.0)	9.6 (8.8–10.4)	6.2 (5.2–7.2)	
unadjusted HR	11.63 (8.64–15.67)	5.27 (4.23–6.57)	2.88 (2.37–3.51)	1.55 (1.30–1.86)	1.00 (-)	<0.0001
adjusted HR ^a^	3.12 (2.25–4.33)	1.77 (1.38–2.27)	1.31 (1.05–1.63)	0.98 (0.81–1.18)	1.00 (-)	<0.0001
**All-cause death**						
number of events	88	246	412	848	243	
event rate (per 1000 P-Y)	102.7 (81.2–124.1)	50.0 (43.7–56.2)	27.4 (24.8–30.0)	15.8 (14.8–16.9)	9.6 (8.4–10.9)	
unadjusted HR	11.02 (8.64–14.07)	5.25 (4.40–6.27)	2.85 (2.43–3.34)	1.64 (1.42–1.89)	1.00 (-)	<0.0001
adjusted HR ^a^	2.96 (2.27–3.86)	1.72 (1.41–2.10)	1.23 (1.03–1.46)	0.99 (0.85–1.15)	1.00 (-)	<0.0001
**Myocardial infarction (fatal or not)**						
number of events	23	62	149	419	176	
event rate (per 1000 P-Y)	27.7 (16.4–39.0)	12.8 (9.6–16.0)	10.0 (8.4–11.7)	7.9 (7.1–8.7)	7.1 (6.0–8.1)	
unadjusted HR	3.92 (2.54–6.06)	1.80 (1.35–2.41)	1.42 (1.14–1.76)	1.12 (0.94–1.33)	1.00 (-)	<0.0001
adjusted HR ^a^	2.73 (1.72–4.33)	1.29 (0.94–1.77)	1.11 (0.87–1.42)	0.98 (0.81–1.18)	1.00 (-)	<0.0001
**Stroke (fatal or not)**						
number of events	10	46	110	248	79	
event rate, n/N	11.8 (4.5–19.1)	9.5 (6.7–12.2)	7.4 (6.0–8.8)	4.7 (4.1–5.3)	3.2 (2.5–3.9)	
unadjusted HR	3.78 (1.96–7.31)	3.02 (2.10–4.35)	2.35 (1.76–3.13)	1.48 (1.15–1.91)	1.00 (-)	<0.0001
adjusted HR ^a^	1.47 (0.74–2.93)	1.25 (0.84–1.87)	1.17 (0.85–1.61)	1.00 (0.76–1.30)	1.00 (-)	0.4193
**Hospital admission for heart failure**						
number of events	34	148	267	619	211	
event rate (per 1000 P-Y)	43.1 (28.6–57.6)	32.0 (26.8–37.1)	18.5 (16.3–20.7)	11.9 (11.0–12.8)	8.6 (7.4–9.8)	
unadjusted HR	5.06 (3.52–7.27)	3.74 (3.03–4.61)	2.16 (1.80–2.58)	1.39 (1.19–1.62)	1.00 (-)	<0.0001
adjusted HR ^a^	2.11 (1.44–3.11)	2.08 (1.65–2.63)	1.36 (1.12–1.66)	1.10 (0.93–1.29)	1.00 (-)	<0.0001

P-Y = patient-years; HR = hazard ratio; 95% CI=95% confidence interval. ^a^ Adjusted for age, sex, geographical region, smoking status, diabetes, body-mass index, treated hypertension, baseline systolic blood pressure, low-density and high-density lipoprotein cholesterol levels, previous myocardial infarction, previous percutaneous coronary intervention, previous coronary artery bypass grafting, number of diseased coronary vessels at baseline, peripheral artery disease at baseline, previous stroke or transient ischemic attack, previous hospital admission for (or symptoms of) heart failure, left ventricular ejection fraction, atrial fibrillation or flutter, and baseline drugs (any antiplatelet, statins, angiotensin-converting enzyme inhibitors, angiotensin-receptor blockers, and beta-blockers).

**Table 3:** Crude and adjusted hazard ratios per 5 units decrease in eGFR below 90 mL/min/1.73 m^2^.

	HR (95% CI) Per 5 Unit Decrease in eGFR (in mL/min/1.73 m^2^)	*p*-Value
**Cardiovascular death**		
unadjusted HR	1.179 (1.161–1.198)	<0.0001
adjusted HR ^a^	1.079 (1.060–1.099)	<0.0001
**All-cause death**		
unadjusted HR	1.171 (1.157–1.186)	<0.0001
adjusted HR ^a^	1.073 (1.057–1.088)	<0.0001
**Myocardial infarction (fatal or not)**		
unadjusted HR	1.060 (1.039–1.081)	<0.0001
adjusted HR ^a^	1.029 (1.005–1.053)	0.0154
**Stroke (fatal or not)**		
unadjusted HR	1.110 (1.083–1.138)	<0.0001
adjusted HR ^a^	1.033 (1.004–1.063)	0.0273
**Hopital admission for heart failure**		
unadjusted HR	1.124 (1.107–1.142)	<0.0001
adjusted HR ^a^	1.061 (1.042–1.080)	<0.0001

Values above 90 mL/min/1.73 m^2^ are truncated at this threshold. HR = hazard ratio; 95% CI = 95% confidence interval. ^a^ Adjusted for age, sex, geographical region, smoking status, diabetes, body-mass index, treated hypertension, baseline systolic blood pressure, low-density and high-density lipoprotein cholesterol levels, previous myocardial infarction, previous percutaneous coronary intervention, previous coronary artery bypass grafting, number of diseased coronary vessels at baseline, peripheral artery disease at baseline, previous stroke or transient ischemic attack, previous hospital admission for (or symptoms of) heart failure, left ventricular ejection fraction, atrial fibrillation or flutter, and baseline drugs (any antiplatelet, statins, angiotensin-converting enzyme inhibitors, angiotensin-receptor blockers, and beta-blockers).

## Supplementary Materials

The following are available online at https://www.mdpi.com/2077-0383/9/1/4/s1.
