# Supplementary material for: Chronic Kidney Disease Has a Graded Association with Death and Cardiovascular Outcomes in Stable Coronary Artery Disease: An Analysis of 21,911 Patients from the CLARIFY Registry
Source: J Clin Med. 2019 Dec 18;9(1):4. doi: 10.3390/jcm9010004 (PMC7019870; doi:10.3390/jcm9010004)
Supplement: Supplementary file 1 [file jcm-09-00004-s001.pdf]

Online Supplement

Chronic Kidney Disease has a graded Association with Death and Cardiovascular Outcomes in Stable Coronary Artery Disease: An Analysis of 21 911 Patients from the CLARIFY registry

Emmanuelle Vidal-Petiot, MD,PhD; Nicola Greenlaw, MSc; Paul R. Kalra, MD; Xavier García-Moll, MD; Jean-Claude Tardif,MD; Ian Ford,PhD; Jose Zamorano, MD; Roberto Ferrari, MD; Michal Tendera, MD; Kim M. Fox, MD; and Philippe Gabriel Steg, MD, on behalf of the CLARIFY investigators

Content

eFigure: Flow diagram of study population 1
eTable: Interaction analyses 2
List of the CLARIFY investigators 3

eFigure: Flow diagram of study population

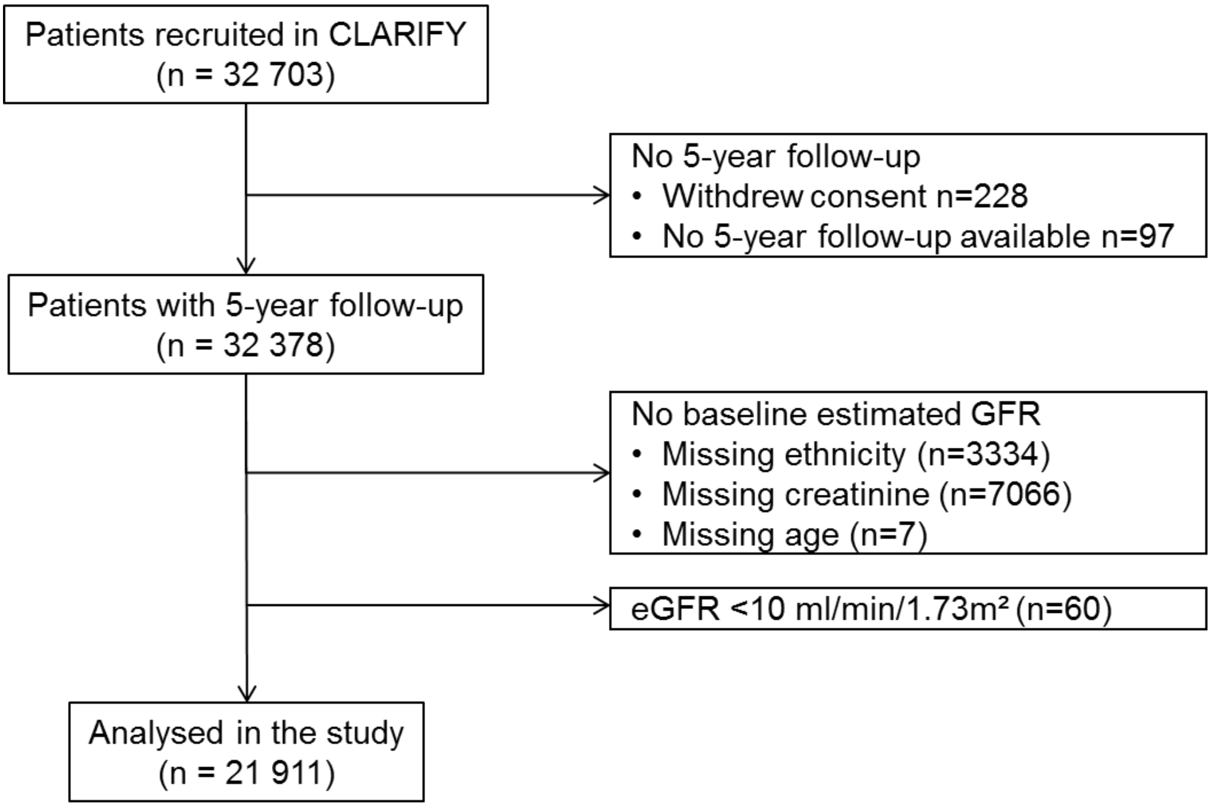

eTable: Interaction analyses

A - P-values for interaction with eGFR (analyzed in 5 categories)			
	**covariate**		
**outcome**	diabetes	hypertension	age
**Cardiovascular death**	0.3179	0.5158	0.5122
**All-cause death**	0.2641	0.7227	0.1985
**Myocardial infarction (fatal or not)**	0.6671	0.2199	0.6911
**Stroke (fatal or not)**	0.6704	0.0543	0.9262
**Hospital admission for heart failure**	0.9943	0.2113	0.1020
B - P-values for interaction with eGFR (analyzed as a continuous variable below 90 mL/min/1.73m²)			
	**covariate**		
**outcome**	diabetes	hypertension	age
**Cardiovascular death**	0.6668	0.8732	0.5590
**All-cause death**	0.3795	0.8543	0.3315
**Myocardial infarction (fatal or not)**	0.2787	0.0090	0.1109
**Stroke (fatal or not)**	0.2330	0.0313	0.1914
**Hospital admission for heart failure**	0.9301	0.2125	0.5114

P-values are indicated for the adjusted model.

No significant interaction was found between CKD and diabetes, treated hypertension, or age when eGFR was analysed in five categories. When eGFR was analysed as a continuous variable, a significant interaction was observed between eGFR and history of hypertension. Indeed, a decreasing eGFR was associated with an increased risk of myocardial infarction and with an increased risk of stroke only in patients with hypertension. Hazard Ratios and 95% Confidence Intervals for the continuous effect of eGFR in models using the total population, those with treated hypertension and those without treated hypertension for each of the outcomes myocardial infarction and stroke are included in the table below.

Outcome	Total Population (N = 21911)*	Patients with Treated Hypertension (N = 15731)*	Patients without Treated Hypertension (N = 6179)*
MI (fatal or non-fatal)			
No. events	829	635	194
Adjusted HR (95% CI)	1.029 (1.005, 1.053)	1.051 (1.026, 1.077)	0.973 (0.921, 1.027)
Stroke (fatal or non-fatal)			
No. events	493	388	105
Adjusted HR (95% CI)	1.033 (1.004, 1.063)	1.073 (1.041, 1.106)	0.979 (0.913, 1.050)

* Results shown are Hazard Ratio and corresponding 95% Confidence Interval for eGFR (per 5 mL/min/1.73m^2 reduction from 90 mL/min/1.73m^2) in the adjusted model for the population noted

List of the CLARIFY investigators

CLARIFY Executive Committee
P G Steg (chair), Paris, France; R Ferrari, Ferrara, Italy; I Ford, Glasgow, UK; K Fox, London, UK; J C Tardif, Montreal, Canada; M Tendera, Katowice, Poland.

CLARIFY Steering Committee
Argentina: Prof. Dr. Fernando José Sokn; **Australia:** Prof. Christopher Reid; **Austria:** Prof.Irene Lang; **Belgium**: Dr. Frank Van den Branden; **Brazil:** Prof. Luis Machado César; Prof. Marco Antonio Mattos; **Brunei**: Dr. Hj. Nazar Luqman; **Bulgaria**: Prof. Assen Goudev; **Canada**: Dr Paul Dorian; **China**: Prof. Dayi Hu; **Czech Republic**: Prof. Petr Widimsky; **Denmark**: Dr. Christian Hassager; **France**: Prof. Nicolas Danchin; **Germany**: Prof. Dr. med. Stefan Kääb; **Greece**: Prof. Panos Vardas; **Gulf Countries**: Dr. Kadhim J. Sulaiman (**Oman**), Dr. Wael Al Mahmeed (**UAE**), Dr. Jassim Al Suwaidi (**Qatar**), Dr. Ibrahim Al Rashdan (**Kuwait**), Dr. Fuad Abdulkader (**Bahrain**); **Hungary**: Prof. Béla Merkely; **India**: Prof. Upendra Kaul; **Ireland**: Prof. Kieran Daly; **Italy**: Prof. Luigi Tavazzi, Prof. Roberto Ferrari; **Korea**: Prof. Yangsoo Jang; **Latvia**: Prof. Andrejs Erglis; **Lithuania**: Prof. Aleksandras Laucevičius; **Malaysia**: Dr. Ahmad Nizar Jamaluddin; **Mexico**: Prof. Marco Alcocer Gamba; **Netherlands**: Dr. Igor I. Tulevski; **Poland**: Prof. Janina Stępińska; **Portugal**: Prof. João Morais; **Romania**: Prof. Dr. Cezar Macarie; **Russia**: Prof Rafael Oganov, Prof Svetlana Shalnova; **Saudi Arabia**: Prof Muayed Al-Zaibag; **Singapore**: Dr. Mak Koon Hou; **Slovakia:** Assoc. Prof. Gabriel Kamensky; **Slovenia**: Prof. Zlatko Fras, Dr. Vojko Kanič; **South Africa**: Prof. Datshana Prakesh Naidoo; **Spain**: Prof. José Luis Zamorano; **Switzerland**: Prof. Hans Rickli, Dr. Andres Jaussi; **Thailand**: Assoc. Prof. Charn Sriratanasathavorn; **UK**: Dr. Paul Kalra; **Ukraine**: Prof. Mykhailo Lutai, Prof. Oleksandr Parkhomenko; **Vietnam**: Prof Lan Viet Nguyen; **West Indies**: Dr. Ronald Henry.

CLARIFY Investigators
Argentina: A. Ahuad Guerrero,M. Basara, F. Belcastro, J.A. Bertarini, C. Cazenave, H. Dreycopp, J. Egido, J. Estrella, D. Garofalo, J. Giordano, H. Lagioia, N. Lago, R. La Greca, L. Lema, N. Lopez Cabanillas, H. Luquez, C. Miller, E. Prada, P. Rodenas, R.G. Schena, G. Suarez, A. Tomatti; **Australia**: D.M. Colquhoun, A. Conradie, S. Cox, D. Cross, R. Fathi, B. Fitzgerald, I. Hamilton-Craig, G. Holt, S. R. Jayasinghe, N. Mai, J. Moolman, R. A. Motyer, K. Phillips, A. Rafter, A. Rahman, A. Rainbird, G. Scalia, A. Taylor, P. West, K. Alford, R. Amor, P. Astridge, B. Bastian, F. Bates, M. M. Doohan, J. Du Plooy, J. C. Ford, L. Kanagaratnam, V. Khoury, R. Parkin, J. Rogers, G. Sceats, A. Waldman, D. Wang, S. Wright, J. Ardill, P. Aylward, J. F. Beltrame, J. Bradley, W. Heddle, M. Joseph, S. Rajendran, S. Varughese, E. Brice, B. Hockings, J. Janssen, A. Kozlowski, J. O'Shea, J. Tan, D. A. Playford, K. Woollard, A. Ajani, G. Barron, N. Better, T. Carruthers, B. Chan, R. Chan, J. Cotroneo, J. T. Counsell, D. S. Eccleston, B. H. R. Forge, A. Hamer, M. Horrigan, V. M. J. Jelinek, R. Lew, D. O'Donnell, F. Panetta, M. Sebastian, P. Shetty, A. Soward, P. Srivastava, N. F. Strathmore, S. Sylivris, G. Szto, V. Veth, T. Yip; **Austria:** R. Badr-Eslam, L. Kleemann, G.Steurer, B. Mörz-Proszowski, F. Auhser, U. Teleky, G. Sepp, A. Beinhauer, D. Kerö, C. Lavicka, T. Perger, V. Hadjiivanov, M. Feldner-Busztin, R. Mika, W. Filip, A. Mahr, J. Toplak, M. G. Millauer, P. Haralambus, K. Walcher, K.H. Karner, E. Ziak, P. Painsipp, U. Frank, A. Suntinger, W. Gritsch, G. Bode, R. Herrmann, R. Raffelsberger, H. Topf, E. Moser, J. Föchterle, T. Honsig, K. Mayr, H. Mayr, R. Kaserbacher, A. Dzien, E. Galehr, M. Felbermayer, R. Schwarz; **Belgium**: R. Amini, H. Appeltants, A. Ballet, C. De Niel, G. Berkenboom, X. Bernard, T. Bouvy, M. Claeys, Y. Dascotte, L. Davin, T. De Backer, A. De Meester, S. De Ridder, P. Dendale, K. Denef, M. Emonts, J. T. M. Geraedts, M. Goethals, J-M. Grégoire, T. Herbots, E. Hoffer, W. H. J. Hutse, P. Lafontaine, P. Lefebvre, H. Lesseliers, G. Odent, A. Pasquet, B. Peperstraete, P. Purnode, A. Rogowsky, M. Rosseel, J-P. Salembier, P. Surmont, A. M. F. Vandeplas, S. Van de Walle, F. Van den Branden, P. Vandergoten, B. G. Vanhauwaert, L. Vanneste, J. Vercammen, D. Verleyen, D. Vermander, C. Weytjens; Brazil: A. Rocha de Lorenzo, A. Felice Castro Issa, B. Mahler Mioto, C. de Brito Vianna, C. Okawabata, E. Giusti Rossi, F. Fernandes, F. Pitella, F. Henpin Yue Cesena, J. F. Monteiro Ferreira, J. F. Junior, L. Tonet, L. Machado Cesar, L. H. Gowdak, M. A. Matos, M. Moretti, R. Vicente Amato, R. Tadeu Munhoz, S. R. Coimbra; **Brazil:** A. Rocha de Lorenzo, A. Felice Castro Issa, B. Mahler Mioto, C. de Brito Vianna, C. Okawabata, E. Giusti Rossi, F. Fernandes, F. Pitella, F. Henpin Yue Cesena, J. F. Monteiro Ferreira, J. F. Junior, L. Tonet, L. Machado Cesar, L. H. Gowdak, M. A. Matos, M. Moretti, R. Vicente Amato, R. Tadeu Munhoz, S. R. Coimbra; **Brunei**: H. N. Luqman; **Bulgaria**: S. Yakovova, M. Mantcheva, V. Mincheva, L. Baurenski, K. Karastanev, V. Yordanova, Y. Peneva; **Canada**: P. Wong, M. Fagan, G. Sabe-Affaki, F. M. Villasenor, W. K. Son, D. E. Manyari, N. Giacomantonio, B. J. Lubelsky, D. Ezekiel, J. C.S. Leong, A. Grover, J. Vavougios, Y. Pesant, A. M. Kushner, M. M. W. Yeung, G. E. Vertes, F. J. Nasser-Sharif, D. Spensieri, A. Roy, T. T. Nguyen, M. Leclair, P. Morra, C. Everton Biglow, J. F. Baril, K. Lai, D. S. Wong, V. Martinho, G. A. Antoniadis, G. R. Searles, D. Rouse, G. Brisson, S. King Wong, R. S. Collette, M. S.

C. Ho, C. Constance, R. Gendreau, G. W. Kellam, T. A. Cieza Lara, H. A. Boyrazian, M. Shamsuzzaman, D. R. Spink Jr., A. P. T.Wong, J. Janes, M. Czarnecka, D. Saulnier, G. Levesque, P. F. Clavette, A. Kokis, T. L. Orenstein-Lyall, A. Shekhar Pandey, J. Robb, G. Verret, W. Czarnecki, F. Perreault, G. Chouinard, G. Lafrance, G. M. Fullerton, P. LeBouthillier, Q. H. Tran, I. Rodriguez Marrero, F. B. Ramadan, P. Talbot, M. A. Fazil, J. Yi-Ming Cha, S. Garg, R. Chehayeb, B. Roy, Y. K. Chan, H. E. Harlos, H. B. Matheson, R. Patel, G. F. Vaz, J. S. Bhatt, E. Liu, T. H. Ashton, H. Sullivan, L. P. Quinn, K. Yared, A. K. Gupta, B. Sullivan, J. Campbell, S. Pallie, H. H. Kim, D. Savard, J. M. Cherry, J. Gold, G. Brouillette, A. Belanger, J. Berlingieri, W. Nisker, G. Boutros, A. I. Bakbak, L. Lasalle; **China**: LL. Chen, MH. Chen, MY. Chen, XP. Chen, YD. Chen, HL. Cong, SF. Ding, JT. Dong, SH. Dong, ZM. Du, LL. Feng, W. Gao, H. Ge, SJ. Ge, T. Guo, Y. Guo, Z. Huang, Y. Huo, HG. Jin, YN. Ke, HW. Li, HM. Li, ZC. Li, YJ. Li, JL. Liu, QL. Liu, SW. Liu, SZ. Lv, W. Miao, GZ. Pan, XD. Pu, ZM. Qian, X. Su, JH. Tao, HP. Wang, JA. Wang, NF. Wang, T. Wang, W. Wang, Y. Wang, M. Wei, SY. Wu, YJ. Wu, LG. Xiong, D. Xu, J. Yan, K. Yang, M. Yang, TL. Yang, XL. Yang, JM. Yu, HS. Zeng, H. Zhang, HQ. Zhang, L. Zhang, SX. Zhou, YL. Zhou. **Czech Republic**: J. Bozkova, J. Carda, S. Dedkova, A. Dufka, J. Fridrich, T. Hodac, R. Jirmar, A. Kadleckova, M. Karlicek, J. Krupicka, J. Kuchar, V. Lavicka, J. Leso, Z. Lorenc, M. Micko, P. Navratil, I. Petrova, P. Povolna, L. Raisova, P. Raska, V. Ravlyk, S. Schlesingerova, E. Smrckova, P. Sternthal, H. Stursova, P. Vymetal, **Denmark**: P. Wiggers, J. Markenvard, L. K. Andersen, L. Frost, J. Refsgaard, S. Strange, K. Egstrup, R. Sykulski, P. Hildebrant, T. Haghfelt, M. Ege, L. Saaby; **France**: S. Cattan, M. Adam-Blanpain, M. Adda, N. Aimouch, L. Ardouin, S. Assouline,A.Aumjaud, C. Barjhoux, R. Baroudi, C. Beaurain, M. A. Bennouna, A. Bernard, C. Bernardeau, E. Blanc, I. Blum-Decary, G. Bodur, C. Boesch, J. Bonal, R. Bonhomme, J. L. Bonnet, J. Bories, M. L. Bourachot, F. Brumelot, M. Brunehaut Petaut, C. Brunschwig, P. Buffet, P. Calmettes, I. Centa, B. Chartier, P. Chemin, F. Chometon, J. Cohen, R. Colin, Y. Cottin, F. Crespo, A. Dabboura, F. David, P. Dehayes, P. Dematteo, O. Dibon, P. Dodemant, V. Dormagen, X. Dreyfus, J. M. Dubois, F, Duclos, M. Ducoudre, O. Duprez, P. Durand, E. Durand, P. Egloff, M. Escande, M. C. Escourrou Berdou, G. Esna Ashari, I Feldmann, J. Ferrieres, E. Foltzer, B. Fontanet, M. Garandeau, T. Garban, S. Geffroy, T. Gillet, S. Godart, P. Gosse, P. Gratia, O. Greiner, A. Gueusquin, E. Guiu, J. M. Guy, S. Haddad, V. Hennebelle, S. Honorat, A. Hourany, G. Hua, P. Jacquier, S. Jean, R. Jeremiasz, P. Kohler, A. Lacroix, M. Leandri, Y. Lemiere, M. Liautard, P Loheac, J. C. Louchart, P Magnus, B, Maheu, H. R. Malaterre, G. Manchet, J. Mantoux, D.Manzi, M. Marachli, M. Maroun, N. Meneveau, E. Messas, J. L. Mougeolle, T. Mouhat, J, J. Muller, M. Naisseh, P. Nocon, D. Onger, A. Ouguoujil, M. Ovize, E. Page, K. Pareathumby, A. Pleskof, P. Poinson, G. Pons, P. Pouderou, J. N. Poujois, V. Probst, F. Prunier, L. Prunier, V. Puel, D. Rechtman, R. Rennert, B. Rijavec, Y. Riou, J. Robert, C. Roche, G. Roul, B. Salaun, B. Saleh, A. Sandalian, M. Sander, A. Schenowitz, A. Silvestre, H. Soleille, S. Tabet, M. Tardy, F. Thomas-Richard, B. Truong, J. Varaldi, H Vial, J. M. Walch, M. Wazana, R. Zeitouni, H. Audibert, F. Alizon, A. Amlaiky,M. Asplanato, C. Baranes, M. Bariaud, F. Bernasconi, P. Bousquet, C. Ceraulo, G. De Geeter, J. Donetti, B. Doucet, J. Doucet, T. Dutoya, D. Ennouchi, M. H. Fallacher, G. Fouquet, V. Fourchard, J. Gdalia, G. Grollier, S. Guerard, P. A. Jeannerat, Y. Jobic, V. Joulie, P. Jourdain, V. Jouve, R. Ketelers, G. Khaznadar, P. Kohan, B. Koujan, B. Lammens, I. Landragin, E. Le Moal, D. M'Bey, F. Maes, S. Maheas Morlet, R. Massabie, D. Meddah, F. X. Meriaux, C. Mestre-Fernandes, P. Meyssonnier, M. Migliore, J. Milewski, J. F. Millet, S. Mingam, P. Nazeyrollas, F. Paganelli, F. Pellerin, F. Petitjean, A. Pinzani, A. Pladys, P. Primot, A. Pucheu, A. Rahali, P. Ravoala, D. Rousson, P. Samama, M. Sardon, R. Silvestri, P. Soskin, X. Tabone, C. Tricot, B. Vaquette, M. Vogel, M. Weingrod, V. Aboyans, R. Amoretti, J. Aubry, P. Berthezene, D. Binet, X. Bonnaud, P. Bonnet, A. Bonny, T. Bouchaya, C. Boureux, J. M. Bourgeois, L. Brottier, B. Cavert, S. Cleron, E. Dechoux, C. Delhomme, J. P. Detienne, J. P. Dubs, B. Faudon, F.Fellous, R. Fressonnet, Y. Garaud, D. Garcia, M. Geneves, J. L. Gleizes, C. Guyetand, B. Hermellin, D. Iovescu, J. P. Kanner, P. Khanoyan, A. Leherissier, A. Maximovitch, B. Merian, P. Messali, Y. Moreau, J. Moyal, L. Payot, L. Petoin Peuch, J. L. Prevot, P. Raymond,D. Relange, S.Reymond, J. F. Robert, H. Rosenstein, J. Schneider, R. Schultz, P. Tanielian, F. Thoin, L. Thomas, P. Touzet, G. Steg, G. Amiel Oster Sauvinet, F. Baylac Domengetroy, K. Chamou, B. Etcheverry, J.L. Farges, J. Y. Fraboulet, M. Goralski, D. Janody, B. Mamez, W. Manlay, F. Paillard, F. Pelier, A. Petit, M. Skonieczny, R. Augarde, J. B. Fournier, S. Liandrat, P. Lim, A. I. Noury, D. Paris, M. Saade, J. M. Stordeur, N. Danchin, M. Pornin, L. Fauchier, M. Galinier, M. A. Balice-Pasquinelli, P. Sosner, S. Yvorra, E. Delcoulx, F. Mouquet, J. E. Poulard, A. Sudre, P. Heno, F. Biausque, M. Guenoun, G. Attia, S. Pouwels, L. Carpentier, E. Verbrugge, C. Ziccarelli, M. Elkohen, J. Tricoire, P. Lang, O. Huttin; **Germany**: B-M. Altevogt, U. Altmann, M. Baar, S. Berrisch-Rahmel, A. Birkenhagen, I. Bläse, R. Blindt, R. Bosch, A. Brattström, H-H. Breuer, M. Castrucci, S. Cicek-Hartvig, R. Cierpka, M. Claus, M. Deissner, M. Drexler, T. Eggeling, G. Eisele, D. Enayat, S. Frickel, S. Gessner, K. Giokoglu, J. Gmehling, F. Goss, P. Grooterhorst, D B. Gysan, R. Haberl, W. Haerer, N. Hassler jun, S. Heinemann-Meerz, F. Henschel, M. Hinrichsen, W. Hofer, A. Hofmeister, G.Hoh, E. Horstkotte, F. Jäger, M. Jeserich, U. Keil, H. Killat, S. Kimmel, M. Kindel, P. Kindler, S. Kleta, J. Könemann, K. König, H. Krause-Allmendinger, K. Kronberg, I. Kruck, V. Männl, A. Meinel, G. Mentz, E. Meyer-Michael, F. Mibach, S. Möller, S.

Muth, E. Nelböck-Huber, D. Ohlmeyer, Z. Özkan-Rashed, C- P. Paulus, S. Perings, J. Placke, C. Raters, N. Reifart, A. Rink, K. Rybak, I. Salecker, K-H. Schermaul, U. Schlesinger-Irsch, E. Schmidt, K-H. Schmitz, N. Schön, T. Schröder, B. Sievers, M. Simon, U. Spengler, M. Speth-Nitschke, A. Stumpp, S. Szabo, J. Taggeselle, A. Tamm, A. Thelemann, C. Thelemann, H. Thümmel, G. Unger, A. Utech, J. Volmar, B. Wauer, G. Wehr, L. Weinrich, R.Weinrich, U. Windstetter, J H. Wirtz, N. Wittlich, P. Ziehn, P. Zündorf; **Gulf Countries**:
Oman: Y. Al Wahshi, P. P. Singh, A. Narayan, F. Al Tamimi, J. Al Yazeedi, M. Ayche, A. Al Lawati, M. Al Dhanki, United Arab Emirates—A. Salustri, T. Salah, M. Y. Tamimi, A. Agrawal, A. Wassef, F. Baslaib, G. Al Radaideh, A. Yusufali, N. Bazargani, Kuwait—M. Akbar, H. Abdel Wahab, S. Abdel Malak, I. Ghaly, H. Hafez, F. Al Kandari, M. Haiba, M. Alanbaei, Qatar—M. M. Gomaa, Bahrain—A. Khalifa; **Greece**: C.Avgerinos, O. Gouli, D. Stergiou, I. Alexopoulos, C. Pappas, I. Petropoulos, G. Chatzioakim, N, Pontikakis, C. Priftis, P. Mpompoth, I. Bourazanis, A Papathanasioy, S. Avlonitis, C. Zakopoulos, G. Koutsimpanis, I. Tsamopoulos, C. Christoforidis, V. Zachos, P. Kalaras, M. Karachaliou, C. Liatas, G. Pournaras, G. Theodorakis, I. Orestis, K. Panisois, E. Chalkiadakis, V. Arfaras, G. Kolios, P. Boutsikos, A. Kotsalos, D. Mitropoulos, A. Samothrakitis, K. Svolis, E. Anastasiou, T. Gkinis, P. Dalampyras, A. Kalampalikis, I. Leontaridis, S. Gabriilidis, I. Konstantinidis, V. Plastiras, P. Tarenidis; **Hungary**: I. Marozsán, I. Édes, I. Czuriga, A. Cziráki, K. Tóth, Á. Dongó, P. Túri, T. Forster, J. Borbola, B. Bachmann, G. Masszi, M. Orbán, G. Gerges, G. Balogh, É. Bajcsi, I. Takács, L. Nagy, B. Kisjós, A. Jánosi, A. Nagy, K. Nagy, A. Büttl, J. Lippai, Zs. Sziegl, Zs. Malkócs, A. Földi, K. Fikker, E. Szabó, R. Forrai, Z. Sebõk, Prof. B. Merkely; **India**: R. Gupta, S. Natarajan, J. Dalal, R. K. Saran, A. Mehta,M. P. Samal, I. A. Khan, T. Ghose, J. P. S. Sawhney, T. Roy, S. Chandra, S. Modi, M. M. Singh, G.Vijayaraghavan, L. Sreenivasa Murthy, S. S. Ramesh, Dr Dayasagar Rao V, M. S. Chenniappan, A. Vadavi, K. Kunhali, K. Srinivasa Reddy, Su. Thillai Vallal, P. Khera, B. Prasad, D. Shukla, A. K. Trivedi, R. Ahuja, J. Rawal, R. Karnik, M.S. Hiremath, D. K. Kumbla, S. R. Shetty, N. S. Chonkar, Late MJuneja, B.K. Goyal; **Ireland**: R. Sheahan, C. Daly, C. Vaughan, S. Fleming, P. Shiels, P. Keelan, T. Kiernan, B. Day, K. Kelly, F. MacNamara, B. Maguire, A. Clifford, A. O'Gara; **Italy**: G. Guardigli, G. Pes, G. Caridi, A. Frattola, B. Doronzo, G. Riccioni, A. Lacchè, F. Massari, S. Orazi, D. Carretta, M. Provvidenza, R. Pedretti, A. Nicolino, S. Felis, V. Pernice, A. Gaglione, P. Gori, P. Martina, V. d'Alessandro, F. Giacomazzi, P. Terrosu, C.Cernetti, R. Antonicelli, G. Ansalone, M. Balbi, C. Tamburino, L. Moretti, S. Tantillo, F. Patriarchi, F. Proietti, G. Sinicropi, G. Maragoni, V. Mallamaci, D. d'Este, P. Azzolini, E. Brscic, S. Bongo, A. Gigantino, G. Perna, M. S. Mayer, C. La Rosa, G. Muscio, V. Scollo, F. Magliari, C. Petrillo Pio, M. Castellari, P. Di Pasquale, Dr Scalzo, F. Saporito, N. Capuano, F. Alitto, N. Marchionni, M. Turiel, A. Bianco, C. Greco, L. Marullo, R. Testa, A. Vicentini, S. Novo, F. La Varra, L. Tavazzi, M. R. Conte, Z. Lazerevic, F. Colivicchi, C. Macchi, J. Dalle Mule, G. Sibilio, A. Achilli, C. Proto ; **Korea**: S.M. Kang, B. K. Koo, S. K. Hong, W. Kim, S. H. Lee, B.S. Yoo, H. S. Seo, H. C. Gwon, D. H. Kang, H. M. Kwon, I. H. Chae, S. J. Oh, J. H. Shin, C. W. Goh, Y.S. Byun, J. H. Zo, T. J. Hong, D. S. Kim, T. J. Cha, J. K. Ryu, Y. J. Kim, J. Y. Hwang, S. H. Hur, M. H. Jeong, S. K. Oh, D. K. Jin, K. T. Jung, J. Y. Rhew, S. Lee, D. W. Jeon, S.J. Yoon, S. H. Kim; **Latvia**: I. Mintale, G. Latkovskis, S. Hansone, N. Rozkova, A. Baika, I. Jasinkevica, S. Abele, I. Laizane, N. Pontaga, V. Ecina, I. Mihailova, A. Kondratovica; **Lithuania**: A. Laucevičius, R. Jurgaitienė, R. Šlapikas, G. Barauskienė, E. Jankauskienė, S. Revienė, D. Zaronskienė, O. B. Šlapikienė, N. Kupstytė, E. Rinkūnienė, R. Steponėnienė, J. Kojelienė, J. Badarienė, V.Dženkevičiūtė, E. Sadauskienė, I. Butkuvienė, R. Stankevičius, R. Paliulionienė, R. Snikytė, R. Mažutavičius; **Malaysia**: A. N. Jamaluddin, A. A. Abdul Rahim, A. K. Mohamed Yusof, K. H. Chee, M. A. Sadiq, S. Ramanaidu, K. H. Sim, T. K. Ong, A. Y. Y. Fong, B. C. Chang, S. K. Chua, Y. L. Cham, N. H. Mohd. Amin, S. K. Tan, N. Z. Khiew, A. Said, C. K. Abdullah, Y.W Cheah, J. Sinnadurai, K. K. H. Lau, C. K. Choor, K. K. Sia, C. C. Ang, J. S. Awtar Singh, M. Z. Abdul Wahab, C. K. Wong, A. K. Ghapar, A. Muthu, K. A. Mahendran, A. H. Jaafar, K. H. Ng, A. I. Ruhani, H. A. R. Tahir, H. Abdul Manap, B. S. K. Ch'ng, E. T. Ch'ng, A. S. Abdullah, O. Ismail, A. S. Sahar, B. B. Abdul Kareem, M. A. Sheikh Abdul Kader, S. K. Ma, K. K. Chan, T. H. Goh, A, Singh, H. B. Liew, C. M. Chu, R. K. M. Bhaskaran, R. P. Shah, K. L. Joseph, H. Noor Hasni, W. K. Ng, G. H. Choo, N. Saaidin, C. K. Yeo, V. M. Lai, Y. C. Lai, M. H. Tay, B. A. Lim; Brunei: H. N. Luqman; **Mexico**: Guillermo Llamas Esperon, J. de Jeús Zuñiga Sedano y America Alvarez, F. Azar Manzur, C. Jerjes Sánchez, J. Cerda Rojas, J. Carrillo Calvillo, F. Petersen Aranguren, C. Martínez Sánchez, A. Álvarez Sangabriel, G. Vieyra, S. González Romero, A. Puente Barragán, F. Redding Escalante, J. Chávez Paez, E. Fernandez Valadez, E. Gaxiola, L. E. Manautou, O. Henne Otero, M. Barrera Bustillos, J. L. Leyva Pons, E. Gómez Álvarez, J. R. Romo Santana, J. Martínez Redding, A. Arias Mendoza, I. Rodríguez Briones, J. de Jesús Rivera Arellano, J. L. Arenas León, M. Alcocer Gamba, E. Alexanderson, M. E. Ruíz Esparza, L. A. Elizondo Sifuentes, J. L. Briseño, E. Sandoval Rodriguez, M. L. Fuantos Delgado, S. Sandoval Navarrete, U. Casas Juarezy, A. Loera Pinales, A. Castro, R. Cue Carpio, E. Rodríguez, G. Rojas, G. Solache, R. Díaz Juárez, R. Baleón, C. Ferreyra Solorio, H. A. Ramírez Reyes, M. López Martínez, M. A. Romero Maldonado, J. Escobedo de la Peña, J. Hilario Jiménez Orozco, F. A. Reyes Cisneros, J. Álvarez Gil, G. Bautista López, M. Odín de los Ríos Ibarra; **Netherlands**: I. I.Tulevski, G. A. Somsen, K. Miedema; **Poland**: I. Chlewicka,P. Brodzicki, T.

Stasiuk, P. Szałkowski, W. Kulig, M. Maliszewski, K. Królicka, J. Zdrojewska, I. Nikodemska, A. Szpak, M. Wrębiak-Trznadel, A. Prokop, M. Szulc, A. Olszewski, W. Kępa, J. Banach, M.Węglarz, A. Gałuszka-Bilińska, A. Królak, E. Cisowska-Drozd, K. Orzechowski, M. Jeżewska, K. Adamaszek, G. Glanowska, T. Pitsch, G. Matuszewska, A. Nowowiejska-Wiewióra, M. Dereń, G. Walawski, M. Sołtysiak, R. Wysocki, G. Jarosiński, A. Drzewiecka, T. Ługowski, A. Jankowska, P. Błaszczak, J. Drozd, E. Łotocka, R. Duchowska, D.Sobczyk, P. Jarmużek, M. Sidor, D. Adamczyk-Kot, J. Sudnik, J. Cygler, I. Skoczylas, B. Poprawa, L. Kisiel, U. Kossowska, B. Sikorska-Buczkowska, K. Modzelewska, B. Demianiuk, W. Streb, T. Mularek-Kubzdela, P. Bogdański, E. Kaźmierczak, R. Zimoląg, J. Lorenc, R. Furtak, A. Regulska, M. Winter, M. Fic, P. Turek, E. Nowicka, W. Bryl, L. Lenartowska, O. Jerzykowska, M. Maćków, W. Gadziński, R. Kacorzyk, D. Zalewska, R. Sadłowski, J. Słaboszewska, M. Gruchała, A. Frankiewicz, J. Walczewska, A. Adamkiewicz-Piejko, R. Chyrek, L. Jankowska; **Portugal**: A. Correia, A. Girão, Á. Herdade, A. Sequeira, A. Tavares E Taveira, A. Gonzaga, A. Ribeiro, A. Albuquerque, A. Fernandes, A. Estriga, A. Rocha De Almeida, A. Lourenço, A. Pereira, A. Faria, B. Carvalho De Moura, C. Camossa, C. Alves, C. Aguiar, C. Rodrigues, E. Wellenkamp, F. Fernandes De Sousa, F. Moreira Pinto, F. Matias, G. Silva Alves, G. Bragança, G. Proença, G. Mariano Pêgo, H. Vinhas, I. Arroja, J. Morais, J. Silva E Sá, J. Vasconcelos, J. Matos, J. Freitas, J. Ferreira, J. Costa, J. Alcaravela, J. Mimoso, J. Antunes, J. Ferreira Dos Santos, J. Nobre Dos santos, J. Fernandes, J. Chambel De Aguiar, J. Moreira, J. Carvalho, J. Forte De Carvalho, J. Calaça, L. Simões, L. Lopes Antunes, L. Soares, L. Semedo, L. Macedo, L. Sargento, L. Basto, L. Rebelo, L. Oliveira, M. Catarino Carvalho, M. Alves Costa, M. C. Gamboa, M. F. Ferrão E Vasconcelos, M. H. Custódio, M. I. Mendonça, M. J. Pinto Vaz, M. Espiga De Macedo, M. Lazaro, M. Martins Oliveira, N. Pelicano, N. Lousada, O. Rodrigues, P. Matos Dias, P. F. Fonseca, P. Ferreira, P. Farto E. Abreu, P. Monteiro, R. Seabra Gomes, R. Carvalho, R. Santos, R. Rosado Soares, S. Baptista, S. Reis Monteiro, V. Gil, V. Sanfins, V. Martins; **Romania**:M. Anghel, C. Arsenescu Georgescu, K. Babes, M. Banu, R. Beyer, I. Bratu, A. Bumbu, R. Capalneanu, O. D. Chioncel, T. Chiscaneanu, R. Christodorescu, N. Cindea Nica, M. Cinteza, S. Coman,M. Constantinescu, E. Craiu, G. A. Dan, D. C. Dan, A. Dan, C. M. David, M. Dorobantu, D. Farcas, V. Firastrau, C. Florescu, A. Ghicu, A. Giuca, R. Grigoriu, D. D. Ionescu, L. C. Iosipescu, M. V. Ivan, D. Lighezan, S. Magheru, M. Magherusan, S. M. Marinescu, A. C. Motoc, R. Musetescu, M. Rau, L. Rotaru H. Rus, O. Sirbu, L. Sorodoc, C.M. Spinu, G. Stanciulescu, C. Statescu, M. Toringhibel, R. Trambitas, N. Trocan, A. Tudose, D. Vinereanu, M. Zagreanu, R. Motomancea, C. Militaru; **Russia**: D. Dymova, N. Semenova, A. Zherebtsova, V. Fedoskin, N. Gurianova, N. Bolotova, V. Knyazeva, T. Spitsina, N. Sytilina, N. Atamanchuk, M. Giorgadze, S. Zarechnova, S. Kutuzova, Y. Sharapova, I. Stelmakh, O. Sinyukova, S. Rostik, L. Evtukhova, L. Sukhanova, T. Makhieva, S. Tereshko, V. Kolesnikov, E. Kochurov, B. Marchenko, S. Nurgalieva, Z. Galeeva, E. Andreicheva, V. Zakirova, L. Baleeva, A. Minsafina, N. Borodina, Y. Arkhipova, T. Krechunova, M. Scherbak, A. Merkhi, N. Aksyutina, O. Ratovskaya, E. Suglobova, Y. Kozhelenko, E. Potapova, G. Poluyanova, N. Naberezhnova, E. Daniels, K. Atueva, L. Tsaryabina, A. Kurekhyan, N. Khishova, E. Dubinina, O. Demina, P. Mochkina, E. Bukanina, S. Tolpygina, Y. Polyanskaya, A. Malysheva, T. Kheliya, A. Serazhim, V. Voronina, Y. Lukina, R. Dubinskaya, N. Dmitrieva,M. Kuzyakina, N. Khartova, N. Bokuchava, E. Smirnova, A. Esenokova, Y. Pavlova, O. Smirnova, P. Astrakhantseva, S. Bykovskaya, O. Charikova, K. Berdnik, T. Karaseva, L. Zhabina, N. Oleinikova, O. Dzhkha, S. Grigoryan, E. Yakovenko, T. Ivaschenko, I. Kiseleva, T, Shokina, M. Novikova, A. Khodanov, L. Popova, L. Latyntseva, O. Kilaberiya, K. Makarenkova, N. Nosova, T. Gerasimova, L. Boikova, N. Sharapova, Y. Kulikova, N. Pasechnaya, E. Bulakhova, S. Kurochkina, I. Bratishko, O. Likhobabina, E. Panova, N. Voronina, N. Bizyaeva, O. Gusev, N. Nevolina, T. Arsentieva, I. Budanova, E. London, Melnikova, A. Khripun, L. Polyaeva, E. Osadchuk, O. Krasnoslobodskaya, N. Yakimova, A. Lugin, Y. Sosnova, E. Il'ina, G. Kositsina, I. Shanina, S. Kostomarova, M. Malgina, M. Omelchenko, I. Gorlova, S. Eidelman, A. Salakhova, B. Bondarenko, R. Sopia, N. Baboshina, N. Eliseeva, F. Tumarov, N. Petrochenko, I. Khudina, N. Arabadzhi, V. Samakhovets, L. Tkhorzhevskaya, T. Sinotova, E. Zherlitsyna, S. Minkin, N. Petrova, Y. Tikhonov, N. Shmakova, V. Abduvalieva,M. Kuzmicheva, L. Nikolaeva, O. Varezhnikova, T. Dmitrieva, E. Mikhailova, Y. Yanina, L. Kapustina, Z. Vazhdaeva, G. Golovina, N. Fedorova, I. Nikolaeva, O. Fillipova, L. Gareeva, F. Tuktarova, N. Khmelevskikh, V. Karnot, M. Golub, I. Surovtseva, V. Kulygina, N. Shelomova, I. Kruglova, I. Pokrovskaya, O. Rodina, L. Polkina, N. Biryukova, E. Filippova, E. Kotova, T. Ignatieva, T. Alekseeva, L. Gruznykh, E. Mozerova, E. Moksyuta, E. Kosachek, N. Srtumilenko, O. Baranova, T. Voronova, L. Bayakhchan, I. Grudtsina, L. Gorshkova, O. Shamsutdinova, M. Getman, I. Gorodilova, N. Karnaukhova, V. Rotenberger, L. Isaeva, G. Lebischak, V. Ryzhkova, E. Usoltseva, D. Mescharekova, E. Tavlueva, E. Mineeva, M. Stikhurova, L. Kosareva, O. Grechishkina, S. Nikishina, A. Ilyukhina, O. Gureeva, I. Soin, S. Erofeev, S. Lebedev, l. Kudryavtsev, E. Gamzatov, N. Maximchuk, L. Grekhova, L. Kolevatova, M. Kazakovtseva, O. Kolesova, L. Zharikova, V. Kukaleva, N. Starostina, I. Grushetskaya, V. Kazachkova, I. Pashentseva, S. Shimonenko, I. Sirazov, A. Chernozemova, O. Golubeva, S. Mingalaeva, E. Zatsarina, D. Kozlov, N. Davydova, O. Larina; **Saudi Arabia**: K.F. Alhabib, A. Hersi, H. Al-Backer, H. AlFaleh, A. Mobeirek, M. Arafah, M. Al-Shamiri,

F. El-Shaer, M. Al Zaibag, M. Bdeir, I. Suliman, A. Mukhtar, H. Omar, A. Jamiel, A. Elkrail, M. Alanazy, M. Habab, K. Ashmak, R. Nourallah; **Singapore**: K. H. Mak, B. Singh, T. S. Chee, C. C. Koo, L. P. Low, V. P. Nair, K. S. Ng, S. S. S. Quek, E. H. M. Tan, A. L. R. Ng, H. H. Chuang; **Slovakia**: G. Kamensky, G. Kaliska, J. Murin, K. Hatalova, L. Gaspar, I. Simkova, J. Dubrava, J. Pjontek, D. Pella, A. Banikova, M. Szentivanyi, F. Kovar, J. Benacka, I. Gonos, F. Fazekas, P. Kycina; **Slovenia**: J. Poles, Z. Fras, A. Pernat, A. Veternik, N. Černič-Šuligoj, M. Kerbev, I. Krajnc, P. Zagožen; **South Africa**: A. Alam, B Brown, B. Luke, E. Variava, R. Nethononda, S. Joubert, P. Matthews, L. Nkombua, V. Antia, D. P. Naidoo, J. Bhayat, S. K. George, N. Ranjith, G. H. M. Vawda, S. Govender, I. Soosiwala, K. Shein, M. Panajatovic, J. Flores, M. S. H. Khan, S. Blignaut, K. Coetzee, L. Burgess, V. Freeman, H. D. Theron; **Spain**: M. A. Arnau, Vives, F. J. Abardía Oliva, P. Aguar Carrascosa, V. Albero Martínez, J. M. Alegret Colomer, E. Alegría Ezquerra, C. A. Almeida Fernández, N. Alvarenga Recalde, A. Alvarez Auñon, P. Alvarez García, C. Amo Fernández, C. Amoros Galito, R. Ancín Viguiristi, M. Aparici Feal, A.Ardiaca Capell, J. Arnedillo Pardo, G. Arquero García, V. Arrarte Esteban, M. Baquero Alonso, P. Barahona Pérez, J. L. Bardají Mayor, V. Barriales Alvarez, A. Batalla Celorio, B. Berzal Martín, D.Bierge Valero, J. Blanco Castiñeiras, F. Bosa Ojeda, C. Botana Penas, H. Brufau Redondo, J. Bruguera Cortada, R. Cabrera Solé, F. Calvo Iglesias, S. Cantabrana Miguel, R. Carrillo Cardoso, M. Casanovas Pié, P. Casas Giménez, E. Castillo Lueña, J. A. Castillo Moreno, M. Castillo Orive, A. Chirivella González, J. M. Chopo Alcubilla, V. Climent Payá, M. A. Cobos Gil, J. L. Colomer Martín, A. Concepción Clemente, R. Cortés Sánchez, D. Cremer Luengo, S. Darnes Soler, J. de Andrés Novales, R. De Castro Aritmendiz, J.de Juan Baguda, M. de los Reyes López, J. L. Delgado Prieto, J. L. Díaz Díaz, C. Escobar Cervantes, J. Ezcurdia Sasieta, L. Facila Rubio, C. Falces Salvador, P. Federico Zaragoza, R. Fernández Alvarez, F. Fernández de la Cigoña, L. A. Fernández Lázaro, L. C. Fernández Léoz, R. Fernández Mouzo, M. Fernández-Valls Gómez, B. Ferreiro Rodríguez, C. Franco Aranda, J. Freire Corzo,J. Fuertes Alonso, J. Fuertes Beneitez, E. Galve Basilio, C. García García, M. J. García Martínez, MJ. García González, S. García Ortego, C. García Pindado, V. García Saavedra, J. García-Moll Marimón, R. Gascueña Rubia, D. Gentille Lorente, H Gervas Pavón, R. Gilabert Gómez, J. J. Gómez Barrado, J. J. Gómez Doblas, M. J. Gómez Martinez, C. González Juanatey, V. González Toda, M. Gonzalvez Ortega, E. Gordillo Higuero, J. Hernández Afonso, D. Herrera Fernández, E. Homs Espinach, A. Idoate Gastearena, M. Irurita Latasa, R. Izquierdo González, M. Jaquet Herter, M. Lagares Carballo, J. A. Lastra Galán, B. Limeres González, M. A. López Aranda, L. López Barreiro, D. López Gómez, A. López Granados, V. López Mouriño, J. L. López-Sendón, M. Luaces Méndez, L. Mainar Latorre, E. Marín Araez, F. Marín Ortuño, A. Martín Santana, J. Martínez Florez, J. Martínez González,J. F. Martínez Rivero, D. Marzal Martín, G. F. Mazzanti Mignaqui, A. Melero Pita, E. Molina Laborda, G. Miñana Escrivá, V. Montagud Saavedra, Mª A. Montero Gaspar, J. Mora Robles, J. Morales González, J. Moreno Arribas, Mª T. Moreno Casquete, C. Moya López, N. Murga Eizagaechevarría, F. Narro García, J. Navarro Manchón, C. Navas Navas, E. Novo García, J. A. Núñez Gamero, A. Ordóñez España, J. A. Ortiz de Murua López, E. Orts Soler, E. Otero Chulian, L. Pastor Torres, A. J. Paule Sánchez, M. A. Paz Bermejo, V. Pedrosa del Moral, G. Peña Pérez, J. Á. Perea Egido, L. Pérez de Isla, S. Pérez Ibiricu, Mª A. Pérez Martínez, M. Pérez Paredes, E. Peris Domingo, AM. Peset Cubero, J. Pinar Sopena, C. Pindado Rodríguez, Mª J. Pinilla Lozano, C. Piñero Ramírez, Y. Porras Ramos, E. Prieto Moriche, F. Ramos Ariznabarreta, M. Rayo Gutiérrez, J. M. Roca Catalán, A. Rodríguez Almodóvar, J. Rodríguez Collado, A. Rodríguez Fernández, J. A. Rodríguez Fernández, J. A. Rodríguez Hernández, I. Rodríguez Tejero, I. Romeo Castillejo, D. Romero Alvira, J. A. Romero Hinojosa, C. Romero Menor, P. Rossi Sevillano, E. C. Rueda Calle, J. Rueda Soriano, P. Ruiz Pérez, T. Sagastagoitia Gorostiza, I. Sainz Hidalgo, M. Sandin Rollán, S. Santaolalla Rodríguez, E. Santas Olmeda, J. L. Santos Iglesias,M. L. Sanz Rodríguez, I. Segura Laborda, S. Serrano García, B. Sevilla Toral, L. Silva Melchor, E. Simarro Martín-Ambrioso, R. Sola Casado, C. Soriano Navarro, Mª I. Soto Ruiz, P. Talavera Calle, E. Teijeira Fernández, P L. Torres Díaz, A. Troncoso Gil, F. Trujillo Berraquero, M. A. Ulecia Martínez, J. Umaran Sánchez, C. Vaticón Herreros, A. Vázquez García, J. L. Vega Barbado, E. Velasco Espejo-Saavedra, T. Vicente Vera, M. Vida Gutiérrez, C. Villar Mariscal, G. Vives Boniato, L. Wu Amen, G. Yanes Bowden, J. C, Yañez Wonenburger, J. L. Zamorano Gómez, J. Zarauza Navarro; **Switzerland**: P. Monnier, A. Jaussi, A. Forclaz, M. Grobéty, L. Schlueter, C. Vuille, C. A. Nacht, D. Evéquoz, S. Ciaroni, F. Dominé, J. Bérubé, H. Rickli, J. Hellermann, R. Koller, G. Bourgeois, R. Engel, C. Niederberger, P. Stadler, M. Gnädinger, C. Schmied, T. Wettstein, P. Hilti, C. A. Chételat, F. Sepulcri, H. Brunner, J. Schindler, M. Kraus; **Thailand**: V. Vivekaphirat, S. Panpunnung, S. Kuanprasert, W. Wongcharoen, A. Phrommintikul, J. Harinasuta, O. Si, V. Chaithiraphan, T. Boonyasirinant,W. Boonyapisit, M. Kittipovanonth, A. Buakhamsri, D. Piyayotai, P. Hutayanon; **UK**: S. Junejo, O. Aiyegbayo, H. Ancliff, C. Bradshaw, R. Cervenak, H. Choi, E. George, I. Gilmour, D. Gough, A. Idrissi-Sbai, J. Ingham, B. Al-Khalidi, A. Liston, J. Mackrell, I. Pattison, R. Ramachandran, N. Ray, G. Reddy, I. Sen, K. Shetty, L. Singh, M. Stanley, A. Wallace, M.Weatherhead, T. Gilbert, G. McCansh, S. Higgins, C. Killeen, I. Cromarty, P. Franklin, E. Pinch, A. Dhesi, C. Dernedde, M. Lawrence, H. Simper, M. Noble, G. Dalton, L. Stevens, P. Berry, C. Hand, R. Oliver, H. Jones, P. Sampson, N. Taylor, R. Grogono, J. Dalrymple, A. Martin, S. Thurston, K. Elsby, M. Vallis, G.

Morrison, C. Lang, A. Watson, A. Thomson, H. Dougall, B. La Hay, L. Compson, A. McCracken, J. Calder, F. Weber, D. Richmond, R. Brownlie, G. Brown, H. MacCowan, A. Heap, M. Perry, L. A. Holden, G. Scott, N. Haldane, S. Hood, I. Cullen, J. Bell P. McNaught, M. Sharif, J. Dunn, D. Hay, S. Ross, R. Shaw, L. Hay, S. Langridge, R. Burns, L. Crawford, A. Kennedy, D. Logan, P. McAlavey, M. Brown, P. Costello, G. McLaren, A. Potter, J. McPherson, M. Drijfhout, J. Finlayson, D. Troup, A. Woodall, J. Pearce, S. Williams, W. Parkar, A. Yusuf, I. Benett, P. Bishop, H. Thomas, I. Caldwell, P. Ormiston, S. Kwok, S. Wright, N. Kanumilli, P. Saul, H. Milligan, I. Wilkinson, A. Vance, N. Paul, C. Paul, I. Shaikh, R. Ellis, N. Vites, R. Steeds, D. Goodwin, A. Aftab, S. Banham, N. Chauhan,M. S. Grocutt, A. Gupte, R. Jordan, B. S. Jheeta, K. Ladha, M. Nazir, R. Pal, R. P. Patel, R. McManus, A. Singal, P. Saunders, A. B. Syed, A. Bahal, H. Dau, D. M. Walker, R. McNeilly, A. Bolidai, N. MacCarthy, D. Lawton, M. Vardhani, G. Sengupta, D. Kinloch, F. Howie, A. Serrano-Garcia, S. E. Paget, R. Till, P. Seal, J. Morrell, T. Maxwell, G. Singh, D. Warden, R. Elias, C. Dixon, R. K. Pandey, V. Challenor, S. Davies, M. Gibbs, A. Gillet, C. Goldie, I. Jarvis, P. Johnson, M. Malden, J. Moore, C. Morton, K. Nehrig, P. Sheringham, G. Wilson, J. Halcox, I. O'Connor, K. Ling, D. Edwards, H. Charles, A. Weatherup, E. Davies, N. Watkins, D. Morgan, R. Davies, A. Lindsay, D. Beacock, R. Balai, P. Kirmond, P. Brindle, C. Bundy, T. Cahill, A. Dayani, P. Eavis, S. Mohr, S. Hayne, C. Krasucki, M. Micheals, I. Orpen, I. Parker, R. Sewell, D. Sharp, A. Smith, A. Stevens, J. Upton, J. Victory, C Wernham, R. Davis, C. Mays, M. Andrews, J. Takhar, C. Travill, P. Choudhury, W. Matta, A. Ihonor, C. O'Dong, S. Rahman, P. Singer, S. Gillam, P. S. Bath, N. Razzaq, O. O'Toole, P. Rowe, H. Williams, P. Kalra, A. Allcock, A. Tucker, V. Sprott, K. Kyd, G. Cunliffe, C. Arden, A. Bateman, G. Kassianos, D. Sinclair, C. Turner, R. Jagathesan, F. Sattar, A. Ashford, A. Chukwu, H. Taylor, R. Pradhan, T. Rundell, R. Howlett, R. Bietzk, R. Patel, M. Myint, M. Partington, F. O'Reilly, M. Baverstock, S. Dixon,M. Tennekoon, N. Brand, P. Haimes, P. Keller, S. Whetstone, R. Davis, C. Mays, M. Andrews, J. Takhar; L. Parker, M. Anscombe, G. Beale, L. Murphy, A. Smith, S. Brown, J. Lindford, A. Serrano, S. Preston, R. Sethi, S. Hutchinson; **Ukraine**: O. Kovyrshyna, V. Rogozhyna,T. Kiver, V. Vasylenko, L. Kucheryava, S. Salimova, V. Alekseenko, O. Gukov, I. Myhailiv, L. Kardashevskaya, O. Prikolota, O. Bashkirtcev, E. Andreev, L. Tkachenko, M. Mospan, V. Batushkin, D. Reshotko; L. Safonova, A. Ogorodnichuk, S. Pustovit, S. Romanov, L. Burlakova, Y. Voloshko, V. Lafarenko, Z. Vlasuk, O. Leshchuk, S. Chushak, V. Koval, O. Stasuk, O. Pogrebna, S. Kornienko, S. Tikhonova,T. Fesenko, T. Kuzmina, O. Ushakov, N. Vechtomova, L. Potapska, I. Illushechkin, E. Kryvenkova, O. Lysunets, O. Tsygankov, L. Bardachenko, L. Voloshyna, V. Ginzburg, L. Franskyavichene, T. Korotich, N. Vyshnevaya, N. Bilous, S. Kulinich, V. Kulik, I. Sadykova, O. Zalyzniak; T. Berezhna, S. Molotyagina; **Vietnam**: L. V. Nguyen, M. H. Pham, H. T. Pham, N. H. Khong, K. B. Do, T. B. LE, P. A. Do, T. C. Do, N. Q. Nguyen, Q. H. Do, K. C. Vu, N. H. Pham, T. H. T. Pham, M. C. Ta, D. P. Phan, T. T. H. Nguyen, T. T. N. Pham, T. L. To, V. T. Le, L. Dang, L. Bui, T. T. H. Pham, H. H. Phan, T. T. H. Bui, T. V. A. Tuong, T. P. Nguyen, T. H. Nguyen, B. K. Nguyen, D. B. Vu, N. S. Pham, T. Q. Do, T. S. Pham, V. D. Dang, D. T. Le, V. C. Do, T. K. L. Nguyen, H. D. Luong, T. Q. Luu, N. V. Pham, T. K. Huynh, N. T. H. Tu, K. A. Ngo, T. T. C. Nguyen, T. T. L. ONG, V. B. Doan, T. B. Kim, T. N. Vo, T. T. T. Tran, T. A. Nguyen, V. D. Tran, A. K. Nguyen, A. C. Tran, M. H. Ngo, N. H. Vu, I. T. Ly, N. P. H. Tran, L. U. P. Tran, T. N. Nguyen, T. H. Tran, P. H. Truong, T. L. Mai, V. S. Hoang, C. M. A. Bui, V. P. Dang, Q. B. Truong, M. P. Vo, V. T. Nguyen, N. H. Chau, T. T. H. Ta, H. N. Dinh, H. Tran, H. K. N. Nguyen; **West Indies**: A. Chung, E. Chung, B. Martina-Hooi, R. Angela, P. Ramoutar, R. Fillet, R. Tilluckdharry, T. Dookie, E. Foster, C. Hart, F. Omardeen, S. Ramphall, C. Lalla, R. Henry, J. Cheng, V. Elliott, H. Falconer, L. Hurlock-Clarke, R. Ishmael, G. Lalljie, K. Lee, A. Liqui-Lung, R. Massay, H. Mohammed, C. Brown, R. Daniel, M. Didier.
